# Functional Reconstitution into Liposomes of Purified Human RhCG Ammonia Channel

**DOI:** 10.1371/journal.pone.0008921

**Published:** 2010-01-28

**Authors:** Isabelle Mouro-Chanteloup, Sylvie Cochet, Mohamed Chami, Sandrine Genetet, Nedjma Zidi-Yahiaoui, Andreas Engel, Yves Colin, Olivier Bertrand, Pierre Ripoche

**Affiliations:** 1 INSERM UMR_S 665, Paris, France; 2 Université Paris Diderot-Paris 7, Paris, France; 3 Institut National de la Transfusion Sanguine, Paris, France; 4 C-CINA, Center for Imaging and Nanoanalytics, E. Müller Institute for Structural Biology, Biozentrum, University of Basel Mattenstrasse 26, Basel, Switzerland; University of Portsmouth, United Kingdom

## Abstract

**Background:**

Rh glycoproteins (RhAG, RhBG, RhCG) are members of the Amt/Mep/Rh family which facilitate movement of ammonium across plasma membranes. Changes in ammonium transport activity following expression of Rh glycoproteins have been described in different heterologous systems such as yeasts, oocytes and eukaryotic cell lines. However, in these complex systems, a potential contribution of endogenous proteins to this function cannot be excluded. To demonstrate that Rh glycoproteins by themselves transport NH_3_, human RhCG was purified to homogeneity and reconstituted into liposomes, giving new insights into its channel functional properties.

**Methodology/Principal Findings:**

An HA-tag introduced in the second extracellular loop of RhCG was used to purify to homogeneity the HA-tagged RhCG glycoprotein from detergent-solubilized recombinant HEK293E cells. Electron microscopy analysis of negatively stained purified RhCG-HA revealed, after image processing, homogeneous particles of 9 nm diameter with a trimeric protein structure. Reconstitution was performed with sphingomyelin, phosphatidylcholine and phosphatidic acid lipids in the presence of the C_12_E_8_ detergent which was subsequently removed by Biobeads. Control of protein incorporation was carried out by freeze-fracture electron microscopy. Particle density in liposomes was a function of the Lipid/Protein ratio. When compared to empty liposomes, ammonium permeability was increased two and three fold in RhCG-proteoliposomes, depending on the Lipid/Protein ratio (1/300 and 1/150, respectively). This strong NH_3_ transport was reversibly inhibited by mercuric and copper salts and exhibited a low Arrhenius activation energy.

**Conclusions/Significance:**

This study allowed the determination of ammonia permeability per RhCG monomer, showing that the apparent Punit_NH3_ (around 1×10^−3^ µm^3^.s^−1^) is close to the permeability measured in HEK293E cells expressing a recombinant human RhCG (1.60×10^−3^ µm^3^.s^−1^), and in human red blood cells endogenously expressing RhAG (2.18×10^−3^ µm^3^.s^−1^). The major finding of this study is that RhCG protein is active as an NH_3_ channel and that this function does not require any protein partner.

## Introduction

While ammonium movement across the plasma membrane is a fundamental process which provides the principal source of nitrogen for microorganisms, it is known, in animals, to be involved in acido-basic regulation in the kidney [1] and can be associated with cytotoxic effects leading, for example to hepatic encephalopathy [2].

The molecular mechanism by which the ammonium crosses the plasma membrane is not completely understood. The similarity of amino-acid-sequences between mammalian Rh (Rhesus) family proteins and ammonium transport proteins of bacteria, fungi, plants and invertebrates [3], [4] suggested that Rh proteins, members of the Amt/Mep/Rh super family, could fulfill the function of ammonium transporter. Human Rh proteins comprise the Rhesus blood group antigens (RhCE and RhD) organized within the erythrocyte membrane as a multimolecular complex including the associated glycoprotein (RhAG) [5]. Two non erythroid members (RhBG and RhCG) are expressed in the connecting tubule and the collecting duct of the mammalian nephron [6], [7], [8]. They are also expressed in a wide variety of extra renal tissues in which ammonium transport is important [9], [10]. Recently, a physiological role of Rhcg in renal ammonium excretion and male fertility was demonstrated in a *Rhcg^−/−^* mouse model [11]. Functional studies which were carried out in different heterologous systems such as yeasts [12], [13], *Xenopus* oocytes [14], [15], [16], [17], [18] and recombinant eukaryotic cells [19], [20], [21] or in red blood cells [22] provided insights into the mechanisms used by Rh glycoproteins for ammonium transport. However, most of these systems contain potential endogenous transporters or acid-base regulating proteins that may interfere with the ammonium transport mediated by the recombinant Rh proteins. Furthermore an uncontrolled parameter of these expression systems may be the membrane NH_3_ permeability depending on the lipid components forming the lipid bilayer.

In previous papers [20], [21], we used the HEK293E expression system which was shown to provide a high level of recombinant RhCG proteins. Using this system, we produced a pool of cells expressing recombinant RhCG protein which contains a double HA-tag in its second extracellular loop. We previously showed that this protein was fully active when compared to untagged proteins [21]. This new tool was used in this study to purify the RhCG-HA protein to homogeneity and to perform functional analysis after reconstitution of this protein into liposomes.

## Results and Discussion

### HA-Tagged RhCG Purification

**Figure 1:**
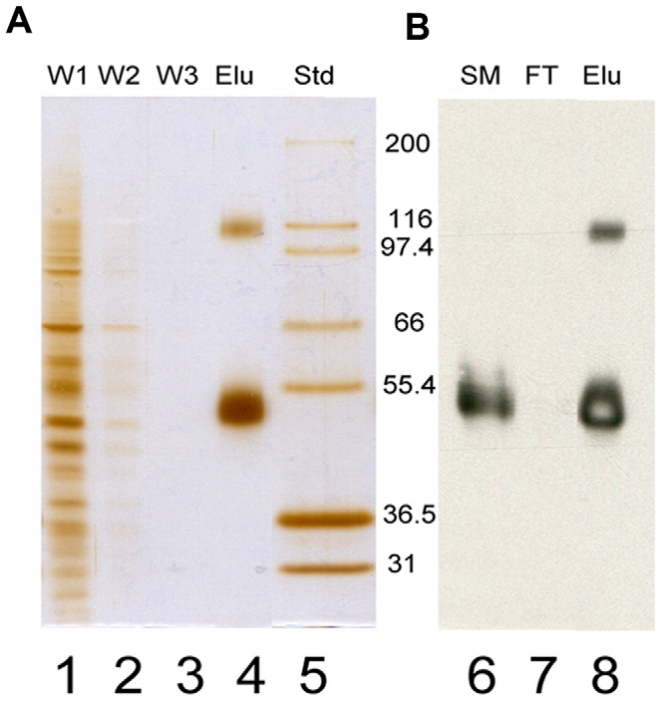
Quality control of RhCG purification. A 10% SDS PAGE was divided in two parts (A and B) after migration. A, Silver staining. lanes 1 to 3: aliquots of the three Washes (W1, W2, W3); lane 4: HA peptide Eluted fraction (Elu); lane 5: molecular weight Standards (Std) from Invitrogen. B, Western blot probed with an anti HA and revealed with Amersham ECL Western blotting detection reagent (GE Healthcare, Buckinghamshire, UK), RhCG can be detected in starting material (SM) on lane 6 and in the HA peptide Eluted fraction (Elu) on line 8 but not in the flow through (FT) on lane 7. Arrows indicate the two bands of 50 kDa and 110 kDa detected by both silver staining and western blot analysis, most likely corresponding to monomeric and oligomeric forms of RhCG.

RhCG was efficiently purified by immunocapture on agarose gel from 2×10^8^ HEK293E cells expressing the recombinant protein. The use of a double HA tag previously introduced in the second extracellular loop of RhCG [21], immuno affinity capture of the tagged protein and affinity elution with the epitope peptide allowed the obtention of RhCG protein of very high purity without exposing it to deleterious buffer conditions. The fractions of different the purification steps, Starting Material (SM) and Flow through (FT) derived from the lysate before and after incubations with anti-HA, the three Washes (W1, W2, W3) and HA peptide Eluted fraction (Elu) were loaded on a sodium dodecyl sulfate-polyacrylamide gel electrophoresis (SDS-PAGE) (**Figure 1**). The purity was determined by the silver stained gel (lane 1 to 4, **Figure 1A**) which revealed a prominent band corresponding to a protein with an apparent molecular weight of 50 kDa. A second band could be detected at 110 kDa. The western blot probed with an anti HA antibody (lanes 6 to 8, **Figure 1B**) showed that the major band corresponds to the RhCG-HA monomer. We assume that second band, which was also detected by the anti-HA antibody could correspond to an oligomeric form of the protein. On the blot, RhCG could not be detected in the flow through (FT), demonstrating that amount of immobilized anti HA was adequate to capture all extracted protein present in the lysate.

Taking into account the RhCG-HA site number per cell (640,000) that was previously determined [21] and the molecular weight of the protein (50,000 g/mol), a theoretical quantity of protein that can be extracted from 2×10^8^ cells was estimated at a weight of 11 µg.

Purified HA-tagged RhCG protein could not be quantified from the absorbance at 280 nm because of the presence of high amounts of HA peptide in the eluate. Therefore, a sample of the purified protein was loaded on an SDS PAGE together with bovine serum albumin (BSA) standards ranging from 39 ng to 444 ng and the gel stained with Coomassie Brilliant Blue was scanned (not shown), showing that approximately 5.1 µg (corresponding to a yield of 50%) of the purified proteins were obtained from 2×10^8^ cells.

### Oligomeric State of RhCG

**Figure 2:**
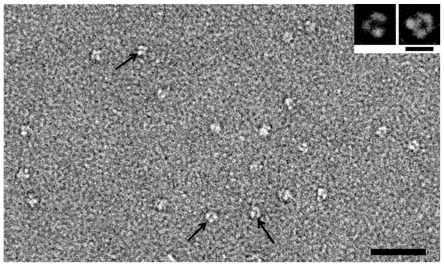
TEM of negatively stained RhCG particles. The electron micrograph reflects the homogeneity of the purified protein. Scale bar is 50 nm. Arrows indicate some top-views of RhCG. In the insert, class averages corresponding to top-views of RhCG are displayed. Scale bar is 10 nm.

Transmission electron microscopy (TEM) was used to ascertain whether the purified transporter is in a monomeric or oligomeric form. Negatively stained purified HA-tagged RhCG revealed particles with a size of 9 nm homogeneously distributed on the carbon film (**Figure 2**). Reference-free alignment and classification of 858 such particles yielded class averages corresponding to different orientations of the molecules on the carbon film. The average of the top view class (See insert in Figure 2) revealed 3 domains. By western blot analysis, an oligomeric form of RhCG was observed despite the presence of SDS (0.1% in the gel and 1% in the sample) but, because of these denaturing conditions, this oligomeric state might be a partial one. The TEM results, suggesting a trimeric form of RhCG, are consistent with homology modelling of the human Rh proteins [23] deduced from structural data on AmtB [24], [25] and with the crystal structures of NeRh50, a bacterial homologue [26], [27] and of human RhCG, recently determined by Gruswitz et al., (PDB 3HD6). This form of the purified RhCG-HA without any detectable residual amount of C12E8 detergent as determined by measurement of the surface tension (data not shown) could be efficiently incorporated into liposomes in order to test its activity.

### RhCG-HA Incorporation into Liposomes

**Figure 3:**
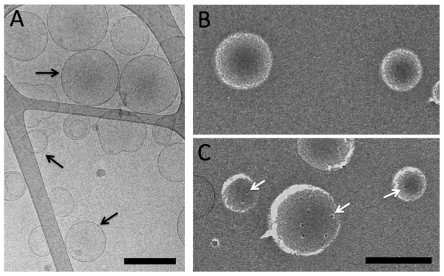
TEM of RhCG incorporated into liposomes. A, Cryo-electron microscopy of vitrified proteoliposomes. Arrows show unilamelar structures. Scale bar is 250 nm. B and C, Freeze fracture electron microscopy of empty liposomes and proteoliposomes (LPR 150) respectively. The arrows indicate RhCG particles. Scale bar is 250 nm.

Cryo-electron microscopy (Cryo-EM) of vitrified samples was applied as a quality control of the proteoliposome reconstitutions. The micrographs showed that the majority of vesicles are unilamellar with a diameter of 100–300 nm. (**Figure 3A**).

Freeze fracture TEM was used to test the incorporation of RhCG-HA into the lipid bilayer and to estimate the number of protein particles per proteoliposome. Aliquots of reconstitutions carried out for transport assays at two different Lipid/Protein Ratios (LPR 150 and 300) were prepared in parallel for freeze fracture. Concave and convex faces of fractured empty liposomes did not exhibit any particles (Figure 3B) while those of proteoliposomes revealed protein particles of 9 nm diameter (**Figure 3C**), which is consistent with the particle size measured by negative staining. This result is in favor of incorporation into liposomes of the RhCG-HA protein in its trimeric form. No aggregates were observed, suggesting that all RhCG molecules were inserted in the proteoliposomes.

The total number of particles per proteoliposomes was measured by counting the particles on the fracture faces. The fracture surfaces of proteoliposomes contained 0–8 and 0–4 particles in reconstitutions at LPR 150 and 300 respectively. Taking into account the diameter of proteoliposomes given by Cryo-EM (175 nm), the average value was corrected. This number was estimated to be 12 and 6 particles corresponding to 36 and 18 monomers (active channels) per proteoliposome at LPR 150 and 300, respectively.

The ratio of the number of channels over the number of vesicles can be calculated by the equation recently reported by Lee et al. [28]: R = (2*4πr^2^*W_RhCG_*M_L_)/(W_L_*σ*M_RhCG_) where r is the assumed average radius of a vesicle (0.08725 µm), W_RhCG_ and W_L_ are micrograms of RhCG and lipid added (5 and 1500 µg respectively), M_L_ is the molecular mass of average lipid molecule (728 Da), σ is the estimated area per lipid molecule (63.E-8 µm^2^), M_RhCG_ is the molecular mass of the RhCG channel trimer (150,000 Da). This ratio was 5 RhCG trimers when a LPR of 300 was used. Assuming that each monomer is an active channel, a maximum of 15 active channels could be present in each vesicles in the tested conditions (LPR = 300). Since Rh channels can function bi-directionally [12], [20], we assume that both orientations of the trimer can be potentially active.

The observed (18) and the calculated (15) numbers of particles are in the same range, allowing a precise determination of the ammonium unit permeability (P_unit_NH_3_) from experimental alkalinisation rate constant values provided by functional assays, as described below.

### Functional Reconstitution of NH_3_ and Methylamine Transport in RhCG-Proteoliposomes

**Figure 4:**
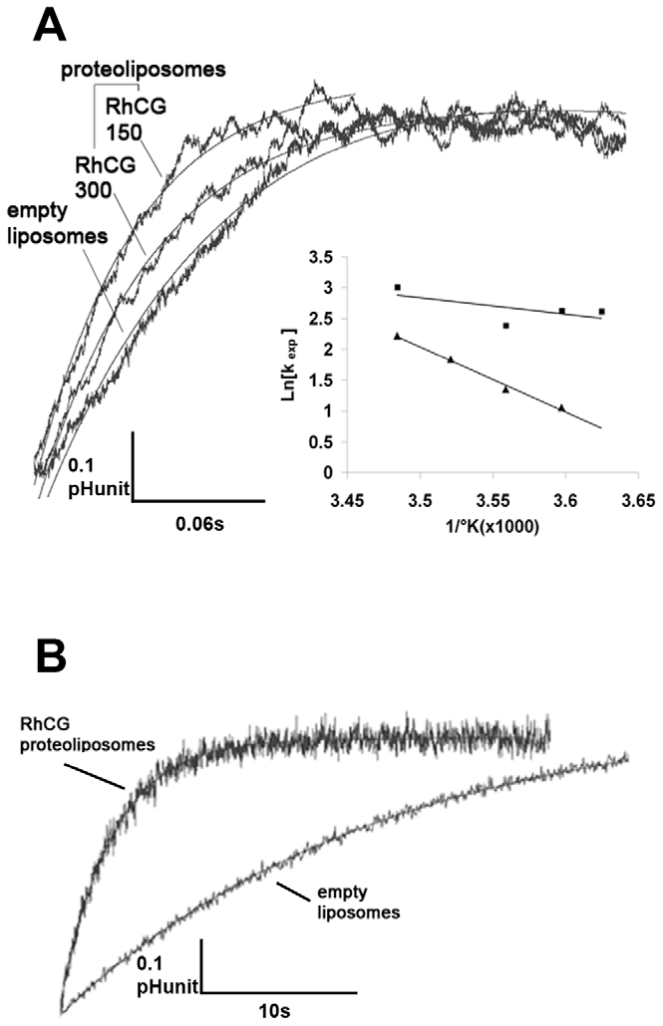
Stopped-flow analysis of ammonia (A) or methylamine (B) fluxes in liposomes and RhCG-proteoliposomes. Individual time courses of fluorescence change were obtained after submitting the empty liposomes and proteoliposomes containing purified RhCG protein to a 10 meq inwardly directed gradient of ammonium (A) or methylammonium (B). In (A) two different LPRs (Lipid/Protein ratio) were used: 150 and 300. Insert in A represents the Arrhenius plots of temperature-dependent ammonia permeation allowing the determination of activation energy (Ea) values from the slopes.

Human HA-tagged RhCG was reconstituted in proteoliposomes at two different LPRs. Alkalinization rate constants of proteoliposomes and empty liposomes were measured by stopped-flow spectrofluorometry. **Figure 4A** shows fluorescence increase kinetics performed at 8°C with liposomes subjected (at t = 0) to an inwardly directed ammonium gradient of 10 meq. The k_exp_ of the exponential curves fitted to the experimental fluorescence variations were 8.3+/−0.48 s^−1^ for empty liposomes and 14.38+/−0.88 s^−1^ and 23.1 s^−1^ (mean of 22 s^−1^ and 24 s^−1^) for proteoliposomes with LPR close to 300 and 150 respectively. Titration carried out with empty liposomes and proteoliposomes showed no significant difference between both types of samples (44 mM/pHunit). Moreover, the size of liposomes was the same for empty liposomes and proteoliposomes, as determined by freeze-fracture study and Cryo-EM of vitrified samples. Therefore, we could compare the two types of liposomes from the analysis of the exponential rate constants, showing an increase of 1.73 to 2.78 fold, depending on the LPR used. Accordingly, apparent P'_NH3_ of the three types of liposomes were 0.46 µm. s^−1^, 0.8 µm. s^−1^ and 1.27 µm. s^−1^, respectively. More interestingly, when the alkalinisation rate constant value of the empty liposome, which corresponds to passive diffusion of NH_3_ through lipids, was subtracted from the values of proteoliposomes, the transport activity was double for proteoliposomes with a LPR of 150 compared to that of proteoliposomes with a LPR of 300. This result clearly showed a correlation between the number of copies incorporated into liposomes and the transport activity deduced from alkalinisation rate constants. Temperature dependence of NH_3_ transport was also determined. Values of Arrhenius activation energy (E_a_) for empty liposomes and for the proteoliposomes were 20.1 kcal mol^−1^ and 5.4 kcal. mol^−1^, respectively (**Figure 4A**, insert). The higher permeability associated to a lower E_a_ in proteoliposomes confirmed that RhCG presents a specific NH_3_ conductance. **Figure 4B** represents an individual curve of alkalinization in presence of an inwardly directed methylammonium (MA) gradient of 10 meq. The alkalinisation rate constant showed a decreased value of 24 fold when compared to that obtained with ammonium (k_exp_MA: 0.25 s^−1^ and k_exp_NH_3_: 6.1 s^−1^; values obtained after subtraction of the component resulting from passive diffusion through lipid). As expected from previous results on HEK293 cells expressing RhCG [20], MA, an analogue with a larger size is less permeant than ammonium through the RhCG channel. As regards the ammonia permeability, this study showed that the apparent Punit_NH3_ per RhCG monomer (around 1×10^−3^ µm^3^.s^−1^) at 8°C was close to the permeability measured in HEK293E cells expressing recombinant human RhCG (1.60×10^−3^ µm^3^.s^−1^) at 15°C [21] and in red blood cells endogenously expressing RhAG (2.18×10^−3^ µm^3^.s^−1^) at 15°C [22].

### Influence of Di-Cations on NH_3_ Transport

The effect on the alkalinisation rate constants of three cations, Thallium, Hg^2+^ and Cu^2+^ was analysed on empty and RhCG-containing liposomes which were submitted to an inwardly directed ammonium gradient of 10 meq at 8°C.

An inhibiting effect of thallium on AmtB has been previously described and is thought to be related to interaction with the ammonium binding site in the external face of the bacterial protein (Javelle et al. 2008). In contrast, thallium has no effect on the ammonium transport activity mediated by RhCG protein, no modification of the alkalinisation rate constant was observed after addition of thallium (0.2 mM) on RhCG proteoliposomes (data not shown). This result is consistent with the absence of an ammonium binding site in the external face of RhCG [21].

**Figure 5:**
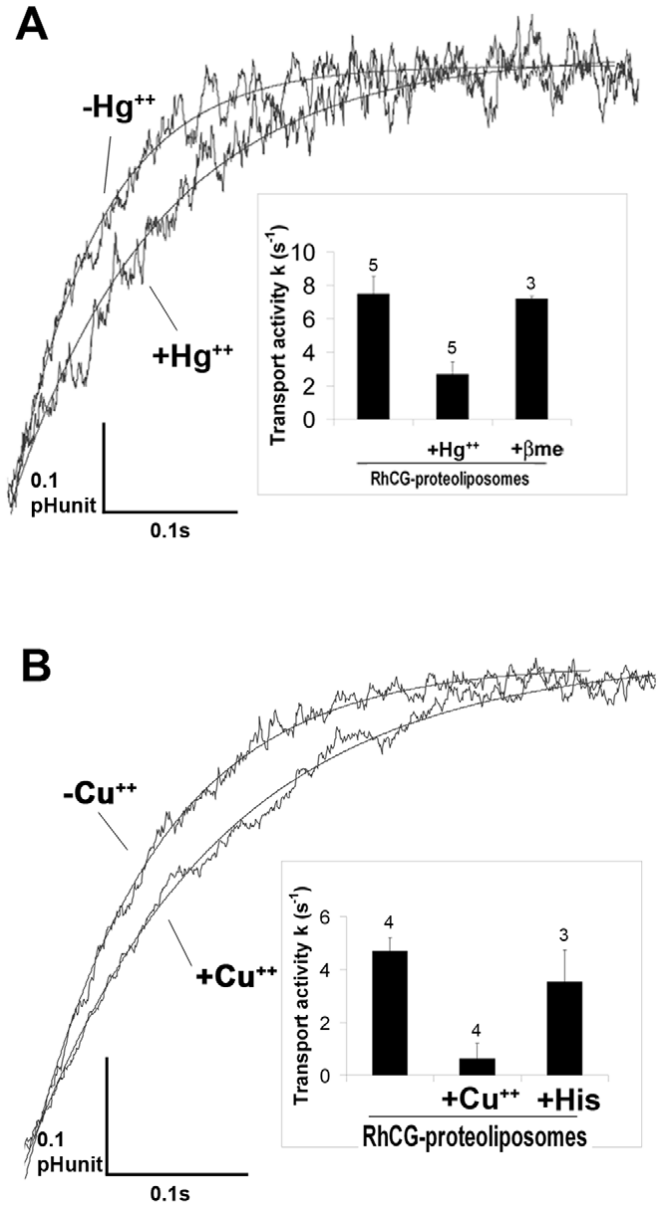
Stopped flow analysis of the influence of divalent cations on NH_3_ fluxes. The effect of Hg^++^ and Cu^++^ on individual time courses of fluorescence change were analysed in RhCG-proteoliposomes after incubation with 0.1 mM HgCl_2_ (A) or 0.21 mM CuSO_4_ (B). For reversion, the β-mercaptoethanol (βme at 5 mM) and the Gly-Gly-His peptide (GGH at 2 mM) were used. Results (means of several independent experiments +/− SEM) regarding the effect of divalent cations (A:+Hg^++^ and B:+Cu^++^) and reversions (A: +βme and B:+GGH) on transport activities (corresponding to alkalinisation rate constants after removal of the constant value of empty liposomes) are reported in inserts. The number of experiments is mentioned above each bar.

A slower alkalinization kinetics was observed after addition of HgCl_2_ (0.1 mM), as assessed by a decrease of the alkalinisation rate constant (k_exp_:10.7 s^−1^ instead of 15.75 s^−1^), while no effect was seen on empty liposomes (k_exp_ (−Hg^2+^):7.5 s^−1^ and k_exp_ (+Hg^2+^):7.5 s^−1^) (**Figure 5A**) After removal of the lipid permeability component, the inhibition of the NH_3_ transport was around 64%, as shown in the insert of **Figure 5A**. This inhibition was reversed by 5 mM βme (β-mercaptoethanol), showing a transport activity recovery of 96%. This result corroborates the experiments carried out in HEK293 cells expressing the recombinant RhCG and showing a reversible inhibition of the NH_3_ transport by Hg^2+^ [20]. Individual or coupled mutations of the four cysteines of RhCG did not render the ammonium transport mediated by RhCG recombinant cells mercury-insensitive (N.Z-Y, unpublished data), indicating that Hg^2+^ does not interact with the cysteine residues of RhCG. A possible interaction between Hg^2+^ and histidines present in the channel pore, as observed with other transporters [29] can be suggested. However, this hypothesis could not be tested, since, of the two mutants of histidines potentially involved, one severely alters cell surface expression of RhCG and the second completely inactivates the ammonium transport function of the protein [21].

Copper is also well-known to form complexes with histidines [30], as recently described for the prion protein in which four histidines coordinate the copper [31]. Incubation of RhCG proteoliposomes with CuSO_4_ (0.2 mM) for 15 min resulted in a decrease of the alkalinisation rate constant (k_exp_:10.56 s^−1^ instead of 14.25 s^−1^), while no significant effect was observed on empty liposomes (−Cu^2+^:8.5 s^−1^ and +Cu^2+^:7.58 s^−1^) (**Figure 5B** **).** After removal of the lipid permeability component, the inhibition of the NH_3_ transport was around 86.7%, (**Figure 5B** **, insert)**. This inhibition was reversed by addition of 2 mM of gly-gly-his peptide (transport activity recovery of 75%).

The effect of divalent cations on ammonium transport by proteoliposomes prepared with purified RhCG definitively proves that RhCG is per se an ammonium transporter. This also confirms that the inhibiting effect of mercuric salt previously reported on RhCG expressed at the surface of HEK293 cells [20] was, at least partially, a direct one. The involvement of the two critical histidines located in the pore of the channel may be suspected but further studies are needed to fully understand the mechanisms of these inhibitions.

### Water Osmotic Permeability of Proteoliposomes

**Figure 6:**
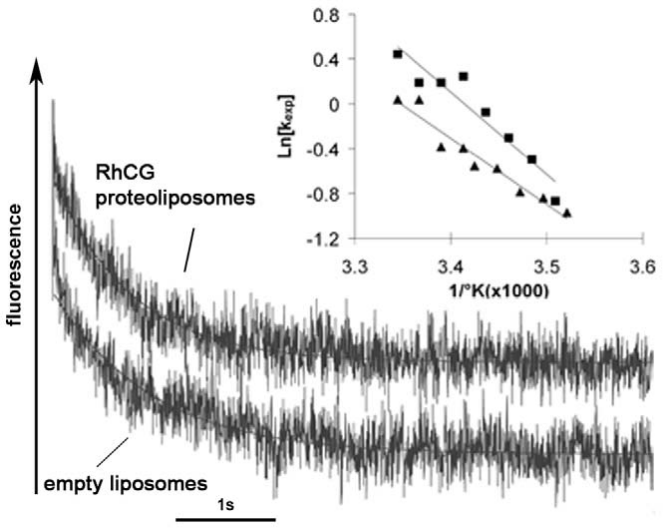
Stopped-flow analysis of water osmotic variations in liposomes and RhCG-proteoliposomes. Individual time courses of fluorescence quenching in RhCG-proteoliposomes were obtained at 20°C with empty liposomes and RhCG-proteoliposomes submitted to a 50 mosmol/kg H_2_O osmotic gradient. Inserts represents the Arrhenius plots of temperature-dependent water permeation allowing the determination of activation energy (Ea) values from the slopes.

Assays performed with recombinant HEK293 cells in which endogeneous putative water channels might be expressed and functional, could not rule out the possibility that the Rh proteins can transport water [21]. Here, water osmotic permeability of liposomes was measured following the fluorescence quenching of carboxyfluorescein. Rate constants of the fluorescence variations were not significantly different between empty and RhCG-containing liposomes since k values were 1.04 s^−1^ and 1.3 s^−1^, respectively. **Figure 6** represents an experimental curve of quenching variations. Liposome water permeability was also determined at several temperatures. Arrhenius activation energy (Ea) values calculated from the slopes of the temperature variations were 14.7 kcal mol^−1^ in empty liposomes and 11.8 kcal mol^−1^ in proteoliposomes. These Ea values were close to that previously determined by Verkman for lipid membranes [32]. These results demonstrate that RhCG is not a significant water channel.

### Conclusion

The present study based on the analysis of ammonium transport after functional reconstitution into liposomes definitively proves that RhCG is active as an NH_3_ channel, which can be reversibly inhibited by mercuric and copper salts. In RhCG-proteoliposomes pH changes measured by spectrofluorometry can be only related to NH_3_ influx following the ammonium gradient. Conversely, in many expression systems previously used to test the activity of Rh proteins, endogeneous irrelevant membrane proteins such as pores or enzymes have the potential to affect ammonium transport assays by modifying acido-basic conditions of the internal or external compartments. Here we show that RhCG protein can be fully active without the contribution of any protein partner. This does not preclude the possibility that, in physiological conditions, RhCG activity could be modulated by other transporters such as V-ATPase (proton pump) or kAE1 (Cl-/bicarbonate exchanger), expressed in the same epithelial cells in the kidney. It is expected that further co-reconstitutions of purified RhCG into liposomes with proteins of interest, as has been described for purified recombinant VGLUT2 and bacterial F-ATPase [33], could provide new insights into the functions of these transporters.

This approach also shows promise for testing the activity and regulation of other members of the Rh family, such as RhAG, the erythroid Rh glycoprotein, which has been shown to belong to a macrocomplex comprising AE1 [34] and ankyrin R [35]. This macrocomplex is thought to function as an integrated CO_2_/O_2_ gas exchange unit (metabolon) in the erythrocyte [34] but the specific role of RhAG regarding this function is not known. Recent evidence that RhAG may induce a cation flux when expressed in *Xenopus* oocytes [36] suggests that sodium could also pass through the RhAG channel. Functional studies of RhAG reconstituted into proteoliposomes may help clarify this issue in order to better define the channel's substrate specificity and understand the direct role of RhAG in normal and pathological conditions.

## Materials and Methods

### Construct of HA-Tagged RhCG Expressing System

The pCEP4-HA-tagged-RhCG vector containing the full length cDNA for human HA-tagged-RhCG was previously described [21]. Briefly, a double HA tag (LYPYDVPDYAGYPYDVPDYADL) was introduced in the second extracellular loop of RhCG. The HA-tagged-RhCG protein was produced after transfection of the kidney cell line HEK293E (Human Embryonic Kidney cell line obtained from Invitrogen) and selection of hygromycin resistant cells was performed as previously described [20]. The expression level was controlled by flow cytometry analysis using a mouse monoclonal anti-HA (HA.11, clone 16B12) antibody purchased from Covance Research products (Berkeley, CA, USA).

### Production and Purification of HA-Tagged RhCG Proteins

The pool of HEK293E cells expressing the HA-tagged RhCG was expanded at 37°C, 5% CO_2_ in Dulbecco's modified Eagle's medium-Glutamax I (GIBCO, Invitrogen) supplemented with 10% fetal calf serum to a final quantity of 2×10^8^ cells. After centrifugations (460 g 10 min at room temperature) and washes in PBS, the cell pellet was suspended in 40 ml of lysis buffer (20 mM Tris pH 8, 0.15 M NaCl, 5 mM EDTA, 1%Triton X100 and a cocktail of protease inhibitors from Roche). The lysate was incubated at 4°C, with gentle mechanical agitation, for one hour. The tubes were centrifuged for one hour at 72,600 g in a Beckman 25.5 Rotor.

For affinity purification, one ml of packed gel, a commercial affinity support (immobilized monoclonal anti-HA antiboby (clone HA7-Agarose, Sigma ref A2095), was equilibrated with the lysis buffer. Gel and cell lysate supernatant were incubated overnight in cold room, with gentle mechanical agitation. The suspension was centrifuged at 12,000 g for 8 minutes. First supernatant was kept and gel was washed by centrifugation first with the lysis buffer (W1) and a second and third time (W2 and W3) with the wash buffer (50 mM K_2_SO_4_, 10 mM Hepes (pH 6.8) containing 0.3% octaethylene glycol monododecyl ether (C_12_E_8_)).

The protein was eluted from the gel by adding 2.5 mg of HA peptide (purchased from NeoMPS, Strasbourg, France) in 600 µl of the wash buffer supplemented with 1% glycerol. The final HA peptide concentration was 3.6 mM. Suspension was incubated for 4 hours at room temperature. After centrifugation at 14,000 g for 10 minutes, the supernatant was carefully collected and kept on ice at 4°C before the proteoliposome reconstitution.

Quality control of the procedure was routinely performed using SDS/PAGE Coomassie and silver staining as well as by western blot probed with anti HA antibody.

### Proteoliposome Reconstitution

Proteoliposomes were prepared according to the step-by-step method [37] using sphingomyelin, phosphatidyl choline and L-α-phosphatidic acid lipids from egg chicken (Avanti) in the ratio: 9∶9∶2, solubilised in C_12_E_8_ (3.7 mM). Proteins were added in the lipid/protein ratios (LPR) 150 and 300 (w/w) in 50 mM K_2_SO_4_, 10 mM Hepes (pH 6.8), and either 0.15 mM 8-hydroxypyrene-1-3-6-trisulfonic acid trisodium salt (pyranine) or 10 mM 6-carboxyfluorescein (CF) as described previously [38]. The detergent was adsorbed with polystyrene beads (Bio-Beads SM2 Adsorbent, Bio-Rad Laboratories) by three successive additions (at ten fold the detergent amount) every hour. After detergent adsorption, external fluorescent probe was removed: for pyranine by filtration across anionic exchange column (AG1-X8 Resin, Bio-Rad Laboratories) and for CF by filtration on gel filtration G25 prepacked columns (GE Healthcare Life Sciences). The residual amount of C12E8 surfactant after adsorption was estimated by the previously described method [39]. Empty liposomes were prepared in a similar manner but RhCG was omitted. Protein insertion was controlled by electron microscopy of freeze-fractured samples.

### Transmission Electron Microscopy (TEM)

#### Negative staining and single particle analysis

A 5 µl aliquot of purified RhCG-HA sample was adsorbed onto glow-discharged 200-mesh carbon-coated copper grids and stained with 2% uranyl-acetate. Images were recorded using Philips CM10 TEM operating at 80 kV. (The micrographs were recorded at a magnification of 52,000× on Kodak SO-163 film). Reference-free alignment was performed on manually selected particles from digitized electron micrographs using EMAN image processing package [40]. Particle projections were classified by multivariate statistical analysis. The class averages with the best signal-to-noise ratio were selected.

#### Cryo-electron microscopy (Cryo-TEM) analysis

A 4 µl aliquot of purified RhCG-HA sample was adsorbed onto holey carbon-coated grid (Lacey support film, Ted Pella, USA), blotted with Whatman 1 filter paper and vitrified into liquid ethane at −178°C. Frozen grids were transferred onto a Philips CM200-FEG electron microscope using a Gatan 626 cryo-holder. Electron micrographs were recorded at an accelerating voltage of 200 kV and a magnification of 50,000×, using a low-dose system (10 e^−^/Å^2^) and keeping the sample at −175°C. Defocus values were −2.5 µm. Micrographs were recorded at 4K CCD camera (Gatan, USA) or on Kodak SO-163 films and then digitized with Heidelberg Primescan 7100 with 4 Å/pixel resolution at the specimen level.

#### Freeze-fracture electron microscopy

Proteoliposomes were centrifuged at 100,000 g in TL100 ultracentrifuge (Beckman) for 30 min at 4°C. The pellet was cryoprotected with glycerol (30% V/V). A small droplet of sample was placed on the copper holder and quenched in liquid propane. The frozen sample was fractured at −125°C in vacuum of about 10^−5^ Pa with a liquid nitrogen-cooled knife in a Balzers 300 freeze-etching unit. The fractured sample was replicated with a 1–1.5 nm deposit of platinum-carbon, and coated with 20 nm carbon film. The Pt/C replica was cleaned with 2% SDS, washed with distilled water, transferred onto copper EM grid and observed with a Philips CM10 electron microscope operating at 80 kV.

#### Measurements of the NH_3_ and methylamine permeability

Reconstitution and assays were done the same day. Experiments were performed at 8°C using 1 ml of RhCG of diluted proteoliposomes (1/3 in 50 mM K_2_SO_4,_ 10 mM Hepes (pH 6.8)). Intravesicular pH (pHi) variations corresponding to ammonium transport were measured in iso-osmotic conditions with a stopped-flow instrument (SFM400, Bio-logic, Grenoble, France), equipped with a cuvette FC-15, 30 µL (dead time in our conditions is 2.6 ms). The excitation wavelength was 463 nm and the emitted light was filtered with a 520 nm cut-on filter. 75 µl of RhCG proteoliposomes were rapidly mixed with the same volume of 40 mM K_2_SO_4,_ 10 mM Hepes (pH 6.8) and 10 mM (NH_4_)_2_SO_4_, or 10 mM methylammonium sulfate, generating an inwardly directed gradient (10 meq) of ammonium or methylammonium. A low experimental temperature (8°C) decreased passive diffusion of NH_3_ through lipids and increased the difference between the rate constants, allowing a better determination of the intravesicular alkalinisations. For inhibition studies, the diluted proteoliposomes were pre-incubated for 15 min in 50 mM K_2_SO_4_, 10 mM Hepes (pH 6.8) buffer containing TlSO_4_ (0.2 mM), HgCl_2_ (0.1 mM) or CuSO_4_ (0.2 mM). For reversion, the βme (5 mM) and the Gly-Gly-His peptide (2 mM) were used.

Data from six to eight time-courses obtained in the different conditions were averaged and fitted to a mono-exponential function using the simplex procedure of the Biokine software (Bio-logic). The equation is: (S)_t_^int^ = (S)^ext^.(1-exp^(-kt)^), where (S)_t_^int^ is the internal concentration of the substrate and (S)^ext^ is the external concentration of the substrate (constant value). k is the rate constant of the exponential. The apparent NH_3_ permeability was calculated from the fluorescence time courses, the size of membrane vesicles according to the equation: P'_NH3_ = k_exp_*ΔpH_i_*_β_*V_0_/S*1/(C_0_−C_i_) [41] where ΔpH_i_ is the intravesicular change value, β is the buffer capacity, V_0_/S is the liposome volume/surface-area ratio, C_0_−C_i_ is the difference in ammonium or methylammonium concentration.

#### Measurements of the water permeability

The water permeability of RhCG proteoliposomes was measured with the carboxyfluorescein quenching method [42] using the SFM400 stopped-flow instrument (Bio-logic, Grenoble, France). The excitation wavelength was 490 nm. 75 µl of RhCG proteoliposome was rapidly mixed with the same volume of hyperosmolar solution (100 mM mannitol in 50 mM K_2_SO_4,_ 10 mM Hepes (pH 6.8)) at 20°C. Because mannitol does not pass through proteoliposome membrane and, since liposomes acted as perfect osmometer, the osmostic gradient (50 mosmol/kg H_2_O) drives the water efflux and the volume change (linearly related to the fluorescence quenching) was analysed.

Data from 5 to 10 time courses were averaged and fitted to a double-exponential function using the simplex procedure of the Biokine software (Bio-logic). The apparent osmotic water permeability P'_f_ was determined as previously described [43]. Pf = k_exp_*V_0_/S*1/V_w_*1/C_out_ where k_exp_ is the first exponential rate constant, V_0_/S is the ratio of liposome osmotic volume to liposome surface area, V_w_ is the molar volume of water (18 cm^3^/mol), C_out_ is the initial concentration of impermeant solute outside the liposome.
